# PCOS-like phenotype in rats after intrauterine EV exposure: puberty and adulthood

**DOI:** 10.1530/EC-26-0315

**Published:** 2026-08-24

**Authors:** Gabriela Rosas, Evelin M Hernández-García, Leonardo Gómez-Emeterio, Rosa Linares, Elizabeth Vieyra, Deyra A Ramírez, Julieta A Espinoza, Andrea Chaparro, Leticia Morales-Ledesma

**Affiliations:** ^1^ Physiology of Reproduction Laboratory, Biology of Reproduction Research Unit, Facultad de Estudios Superiores Zaragoza, Universidad Nacional Autónoma de México, Mexico City, Mexico; ^2^ Endocrinology Laboratory-UIBR, Facultad de Estudios Superiores Zaragoza, Universidad Nacional Autónoma de México, Mexico City, Mexico; ^3^ Medical Surgeon Career, Facultad de Estudios Superiores Zaragoza, Universidad Nacional Autónoma de México, Mexico City, Mexico; ^4^ Neuroendocrinology of Reproduction Research Laboratory-UIBR, Facultad de Estudios Superiores Zaragoza, Universidad Nacional Autónoma de México, Mexico City, Mexico; ^5^ Nursing Career, Facultad de Estudios Superiores Zaragoza, Universidad Nacional Autónoma de México, Mexico City, Mexico; ^6^ Facultad de Estudios Superiores Zaragoza Campus III, Universidad Nacional Autónoma de México, Ex Fábrica de San Manuel 8-A, Comunidad de San Miguel Contla, Mexico City, Mexico

**Keywords:** polycystic ovary syndrome (PCOS), intrauterine exposure, estradiol valerate, folliculogenesis, anovulation, hyperandrogenism

## Abstract

Polycystic ovary syndrome (PCOS) is the most common endocrinopathy affecting female fertility. It has been hypothesized that PCOS has its origins during intrauterine life, where prenatal exposure to endocrine-disrupting environments during critical developmental windows induces developmental disruptions, leading to long-term reproductive dysfunction. While prenatal androgen exposure is a well-established factor, the role of estrogenic imbalance remains critical, yet less analyzed. Estradiol valerate (EV) is widely used to induce PCOS-like phenotypes; however, its potential role in disrupting early development and altering postnatal reproductive function remains unknown. This study evaluated whether prenatal exposure to EV results in PCOS-like reproductive alterations that manifest at puberty or adulthood in rats. For this purpose, gravid rats were subcutaneously injected with EV or sesame oil (Vh) on gestational day 18. Offspring were euthanized at puberty or adult stage, in estrus. Compared with the Vh group, EV-exposed offspring exhibited early-onset reproductive disruptions during puberty, including precocious vaginal opening, estrous acyclicity, low testosterone levels, and a decreased ovulatory response accompanied by precyst formation. In adult life, these alterations had progressed into a PCOS-like phenotype, characterized by body weight gain, increased testosterone levels, acyclicity, anovulation, and the presence of follicular cysts. These findings indicate that prenatal exposure to EV is sufficient to induce a progressive reproductive phenotype that resembles PCOS in adulthood.

## Introduction

Polycystic ovary syndrome (PCOS) is the most prevalent endocrine–metabolic disorder among women of reproductive age, with a global prevalence of 11–13% (1). According to the latest international evidence-based guideline for the assessment and management of polycystic ovary syndrome, a woman must present at least two of the following characteristics to be diagnosed with the syndrome: i) clinical/biochemical hyperandrogenism, with high total testosterone levels, an increase in the free androgen index, and/or high bioavailable testosterone levels; ii) ovarian dysfunction, with irregular or absent menstrual cycles; and iii) polycystic ovarian morphology, with multiple small antral follicles, enlarged ovarian volume, and/or high anti-Müllerian hormone levels (2).

In addition to these criteria, PCOS is frequently associated with a hormonal dysregulation, including elevated estrogen levels and an altered gonadotropin ratio – typically characterized by increased luteinizing hormone (LH) and decreased or normal follicle-stimulating hormone (FSH) levels. Furthermore, the syndrome often encompasses a broad spectrum of metabolic disturbances, such as insulin resistance, dysglycemia, dyslipidemia, overweight, obesity, and metabolic syndrome (1, 3).

The etiology of PCOS is multifactorial, with emerging evidence suggesting that environment and lifestyle play fundamental roles. A critical factor in the development of this pathophysiology is linked to its intrauterine origin, where physiological and structural modifications of tissues occur in response to early stimuli during sensitive developmental windows, subsequently manifesting as pathologies in postnatal life (4, 5, 6, 7). Among these early stimuli, endocrine-disrupting chemicals (EDCs) are of particular concern. The World Health Organization defines EDCs as exogenous substances or chemical mixtures that alter the functions of the endocrine system, consequently causing adverse health effects in an intact organism, its progeny, or sub-populations. By interfering with endocrine homeostasis, EDCs disrupt reproductive and metabolic health in both sexes (6, 7).

The impact of such endocrine disruption is closely mirrored in clinical observations of pathological pregnancies. Studies have reported that women with PCOS who become pregnant exhibit high androgen levels during gestation (8). The placentas of women with PCOS show increased levels of the enzyme 3β-hydroxysteroid dehydrogenase type I (responsible for converting dehydroepiandrosterone into androstenedione), alongside a decrease in P450 aromatase activity, which catalyzes the biosynthesis of estrogens from androgens, contributing to hyperandrogenism (9). Furthermore, daughters of women with PCOS exhibit hallmarks of the syndrome upon reaching puberty, such as elevated LH and testosterone levels, increased ovarian volume, and hyperinsulinemia (10). This clinical evidence suggests a high vulnerability of the fetal environment to endocrine imbalances, where EDCs may act as external triggers by disrupting enzymatic pathways and the steroidal milieu during critical windows of development.

Experimental models have been utilized to analyze the effects of developmental disruptions following hormonal exposure during critical developmental windows. In rhesus monkeys, prenatal exposure to high testosterone levels results in menstrual cycle disruption, increased LH secretion, anovulation, and ovarian enlargement at puberty (11). Similarly, in sheep androgenized with testosterone from days 30 to 90 of gestation, offspring exhibit low birth weight, precocious puberty, anovulatory condition, estrous acyclicity, and diminished ovarian reserve accompanied by increased follicular recruitment (12).

In addition to androgen-based models, the impact of prenatal estrogenization has also been investigated. In mice, exposure to diethylstilbestrol (DES) from gestational days 9 to 16 leads to oviductal malformations and the development of ovarian cysts by the fifth week of postnatal life (13). Mouse offspring exposed to ethinylestradiol from gestational days 11 to 17 exhibit vaginal epithelial cornification, endometrial hyperplasia, increased follicular atresia, and ovarian cysts during adulthood (14).

Estradiol valerate (EV) is a long-acting estrogen (with a half-life of 15 days) frequently used in rat models to induce PCOS-like reproductive alterations. A single dose of EV has been shown to disrupt the estrous cycle, induce anovulation and hyperandrogenism and lead to ovarian cyst formation (15, 16, 17, 18, 19, 20). Despite its established efficacy in prepubertal and adult models, it remains unknown whether prenatal exposure to EV disrupts intrauterine development, predisposing offspring to PCOS-like traits in postnatal life. Therefore, the present study evaluated whether exposure of female fetuses to EV on gestational day 18 results in reproductive alterations consistent with the PCOS phenotype during puberty or adulthood.

## Materials and methods

### Study design

The experimental protocol was approved by the Research Ethics Committee of the Facultad de Estudios Superiores Zaragoza, Universidad Nacional Autónoma de México (FES-Zaragoza, UNAM; Protocol No. FESZ/CEI/4/3/1/05/24). All experimental procedures were carried out in accordance with the specifications established by the Norma Oficial Mexicana NOM-062-ZOO-1999, technical specifications for the production, care, and use of laboratory animals. Every effort was made to minimize the number of rats used and their suffering.

Female and male rats of the CIIZ-V strain from our in-house colony were housed under controlled temperature (22 ± 2°C) and a photoperiod of 14h light:10 h darkness(lights on at 05:00 h).

### Mating and pregnant females

Sexually mature nulliparous female rats (three months of age) were used. The estrous cycle was monitored through daily cytological examination of vaginal smears. On the day of proestrus, two female rats were housed overnight with a male rat of proven fertility. The following morning, the presence of a vaginal sperm plug or the observation of spermatozoa in the vaginal smear was considered indicative of successful mating and designated as gestation day zero (GD 0).

Pregnant rats were individually housed and randomly assigned to the experimental groups. On gestational day 18 (GD 18), the rats received a single subcutaneous (s.c.) injection of either 0.1 mL of sesame oil (vehicle, Vh; Sigma Chemical Co., USA) or 2.0 mg of EV (Sigma Chemical Co., USA) dissolved in 0.1 mL of Vh. GD 18 was selected because it represents a critical developmental window for fetal rat ovarian innervation. While the sympathetic nervous system develops rapidly at GD 17, catecholaminergic fibers only become fully visible by GD 19 (21, 22). Therefore, GD 18 was selected to evaluate whether an estrogenic insult occurring during the onset of ovarian neural integration is associated with the subsequent development of reproductive dysfunction in the offspring. The 2.0 mg dose of EV was selected as a robust pharmacological agent to reliably induce PCOS-like characteristics in rats, such as hyperandrogenism, chronic anovulation, and follicular cysts, distinct from low-dose environmental endocrine disruption protocols (15, 16, 17, 18, 19, 20). The offspring from dams injected with Vh served as the control group, while those from EV-injected dams constituted the experimental group. All pregnant female rats were monitored daily until delivery, which typically occurred on GD 21 or GD 22.

### Offspring and experimental groups

The day of birth was designated as postnatal day 0 (PND 0). On PND 1, to eliminate litter-size-dependent variations in maternal care and postnatal growth, the pups were sexed and standardized to a uniform density of five or six female rats and one male rat per dam, ensuring uniform nutrition and a homogeneous developmental environment. Offspring had free access to their dams until weaning on PND 24. Subsequently, they were provided free access to tap water and a standard pellet diet (Purina S.A., México) until euthanasia.

Offspring were maintained in cages according to their maternal origin and assigned as whole-litter units to their respective experimental endpoints (puberty or adulthood). Notably, when two dams gave birth on the same day, a cross-fostering approach was implemented by mixing the female offspring from both mothers to maximize biological variability within those cages.

Due to the low maternal yield and high gestational mortality induced by prenatal EV exposure, biological replicates within each sub-cohort otherwise include female littermates; this litter-dependent design was implemented to preserve maternal-social stability within cages during the post-weaning developmental period.

Given that injection of EV has been shown to advance puberty onset (19, 20, 23), vaginal opening (VO) was monitored daily, starting on PND 14 for the EV-exposed group and on PND 30 for the vehicle (Vh) control group.

Once vaginal canalization occurred, daily vaginal smears were performed to determine the day of the first vaginal estrus (FVE). A subset of animals was euthanized upon reaching FVE (*n* = 11 per group), while the remaining animals were assigned to the adulthood group (*n* = 11 for Vh and *n* = 12 for EV). For those groups assigned to adulthood, vaginal smears were taken for two consecutive cycles (eight days) immediately after VO and again starting at PND 60 for an additional eight-day period. These rats were euthanized between PND 68 and 72, when they presented an estrus vaginal, to allow for the established 56-day window required for follicular cyst development (15). All animals were euthanized between 10:00 h and 12:00 h.

### Necropsy procedures

Vh- and EV-exposed offspring were weighed and euthanized by decapitation, either upon reaching FVE or in adulthood, according to their assigned experimental group. Trunk blood was collected, allowed to clot at room temperature for 30 min, and centrifuged at 3,500 r.p.m. for 15 min. The serum was separated, aliquoted, and stored at −20°C until the steroid hormones were quantified.

The ovaries and uterus were dissected and weighed using an analytical balance. The oviducts were separated from the ovaries, and then, under a stereoscopic microscope (Nikon, model SMZ800, Japan) and with a pair of dissection needles, the ampulla was identified and opened to count the number of ova shed, following established laboratory protocols (20).

### Ovarian morphometry

The ovaries of three randomly selected rats from each experimental group (six ovaries per group) were removed, cleared of adherent fat, and fixed in Bouin’s solution for 24 h. Tissues were dehydrated in a graded series of alcohol and chloroform and then embedded in Paraplast. Serial sections (10 μm thick) were mounted and stained with hematoxylin–eosin. Morphometric analysis was conducted using a binocular microscope (Nikon, Model Labophot-2).

Follicular diameter was determined by using a calibrated ocular micrometer, only in those follicles whose oocyte presented a well-defined nucleus and nucleolus. Measurements were taken from basement membrane to basement membrane; the mean diameter was calculated by averaging the maximum diameter and the diameter at a right angle (24). Follicles were classified by size as follows: small (90–390 μm), medium (391–500 μm), and large/preovulatory (>501 μm) (25).

Follicles were further categorized as healthy, atretic, precysts, or cysts. Follicles were considered atretic when they presented at least one of the following characteristics: theca cell hypertrophy, pyknotic granulosa cell nuclei, desquamation of these into the follicular antrum, or detachment of the cumulus–oocyte complex (24, 25, 26). Precysts were defined as large follicles with a broad antral cavity, presence or absence of an oocyte, four to five granulosa cell layers, an apparently normal thecal layer, and with invaginations and evaginations of the follicular wall. Follicular cysts were identified by a wide antral cavity, theca hyperplasia, a reduced granulosa layer, and the absence of an oocyte (15, 18).

### Hormone assay

Serum progesterone levels were quantified using a chemiluminescent enzyme immunoassay (CLEIA; Siemens, USA; Cat# LKPW1, RRID:AB_2800399), according to the manufacturer’s instructions. The progesterone levels were expressed in ng/mL. The values of the intra- and inter-assay coefficients of variation were 5.96 and 7.55%, respectively.

Serum testosterone and estradiol levels were measured using ELISA kits, following the protocol provided by the manufacturer (DRG Instruments GmbH, Germany; Cat# EIA-1559, RRID:AB_2885166 for testosterone; and Cat# EIA-4399, RRID:AB_2848162 for estradiol). The levels for both hormones were expressed in pg/mL. The intra- and inter-assay coefficients of variation were 6.42 and 7.32% for testosterone and 6.4 and 8.8% for estradiol, respectively.

### Statistical analysis

Parametric data are expressed as mean ± standard error of the mean (SEM), while non-parametric data are presented as median and interquartile range (25–75th percentiles). All statistical analyses were performed using GraphPad Prism software (v10.3.1; USA). Data normality was assessed using the Shapiro–Wilk test. Body weight (BW), relative weights of the ovaries and uterus, and serum steroid hormone levels were analyzed using Student’s *t*-test. The age at VO and FVE, the number of ova shed, and follicular counts (total, healthy, atretic, precysts and cysts, and size-based classifications) were analyzed using the Mann–Whitney *U* test. The ovulation rate (the number of ovulating animals/the total number of animals) was analyzed using Fisher’s exact test. A value of *P* ≤ 0.05 was considered statistically significant.

## Results

### Puberty onset

Female offspring prenatally exposed to EV showed an advancement in the age of VO (EV: 35 (34–38) vs Vh: 38 (37–41) days; *P* ≤ 0.05) and FVE (EV: 35 (34–38) vs Vh: 38 (37.5–41) days; *P* ≤ 0.05, Mann–Whitney *U* test), compared with the Vh group. This early onset of puberty was consistently observed in both the group euthanized at puberty and the group assigned in adulthood.

### Estrus cycle

**Figure 1:**
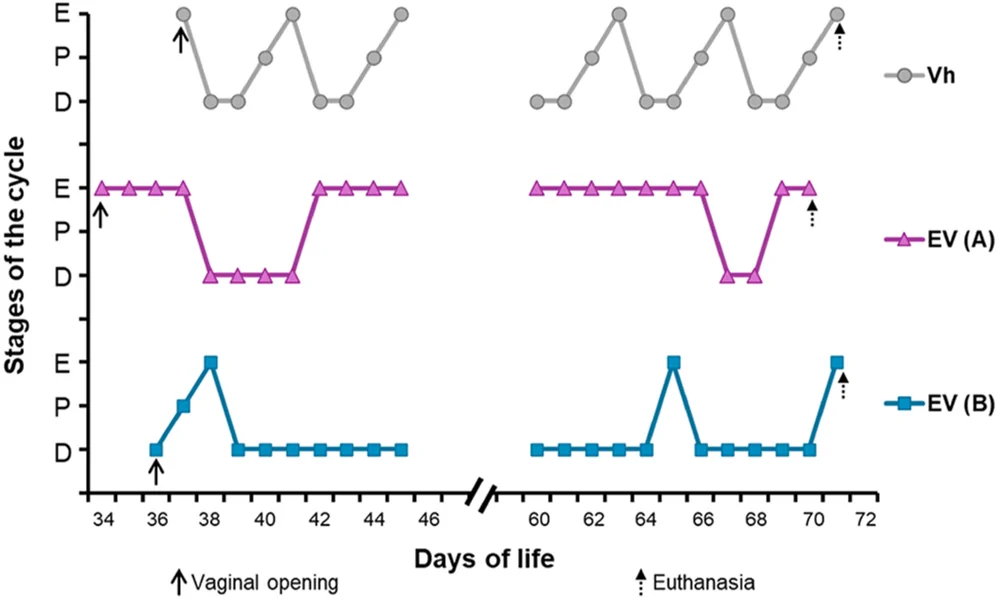
Estrous cycle in prenatally estrogenized rats. Representative cycle pattern of offspring exposed on GD 18 to vehicle (Vh) or estradiol valerate (EV) and euthanized at adulthood during the estrus stage. D, diestrus; P, proestrus; E, estrus. (A) Estrous cycle of EV-exposed animals exhibiting persistent estrus. (B) Estrous cycle of EV-exposed animals exhibiting prolonged diestrus. *n* = 11 for the Vh group and *n* = 12 for the EV group.

Female offspring in the Vh group showed normal estrous cycle patterns at both puberty and adulthood. In contrast, those prenatally exposed to EV showed disrupted estrous cyclicity throughout their development, characterized by persistent estrus (observed in 54.5% of animals) or prolonged diestrus (observed in 45.5% of animals) (Fig. 1).

### Ovulatory response

**Figure 2:**
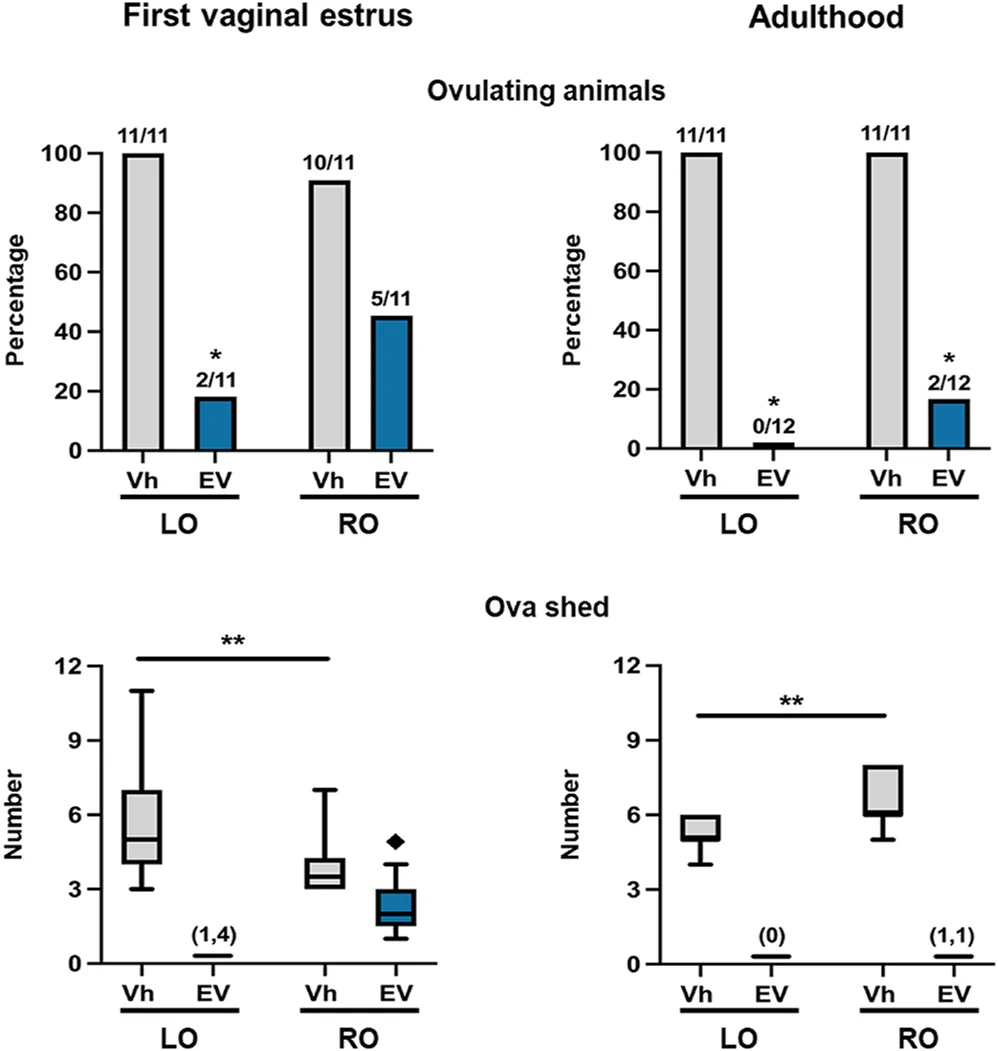
Ovulatory response in prenatally estrogenized rats. Percentage of ovulating animals and median and interquartile range (25–75th percentiles) of the number of ova shed by the left (LO) or right ovary (RO) of offspring exposed on GD 18 to vehicle (Vh) or estradiol valerate (EV) and euthanized at first vaginal estrus (FVE) or in adulthood, in estrus. FVE group (*n* = 11 per group of Vh and EV). Adulthood group (*n* = 11 for Vh and *n* = 12 for EV). The numbers in parentheses represent the absolute number of ova shed by each ovulating animal within the corresponding EV group. *P ≤ 0.05 vs their respective Vh group (Fisher’s exact test). **P ≤ 0.05 vs Vh LO group; **◆** *P* ≤ 0.05 vs Vh RO group (Mann–Whitney *U* test).

All Vh-exposed female rats euthanized at FVE ovulated (left ovary, LO: 100%; right ovary, RO: 91%), although the number of ova shed by the RO was lower than by the LO. In contrast, EV exposure reduced ovulatory response at FVE, where only 20% of these animals ovulated by the left ovary (2 of 11), with a low quota of 1 and 4 oocytes, while by the RO, there was a similarly blunted response, with a significant reduction in the number of ova shed compared with Vh (Fig. 2).

By adulthood, 100% of Vh animals continued to ovulate, with the RO releasing a greater number of ova than the LO. Conversely, EV-exposed offspring exhibited a blockage of ovulation in the LO, while only two animals ovulated a single oocyte from the RO (Fig. 2).

### Ovarian histology

**Figure 3:**
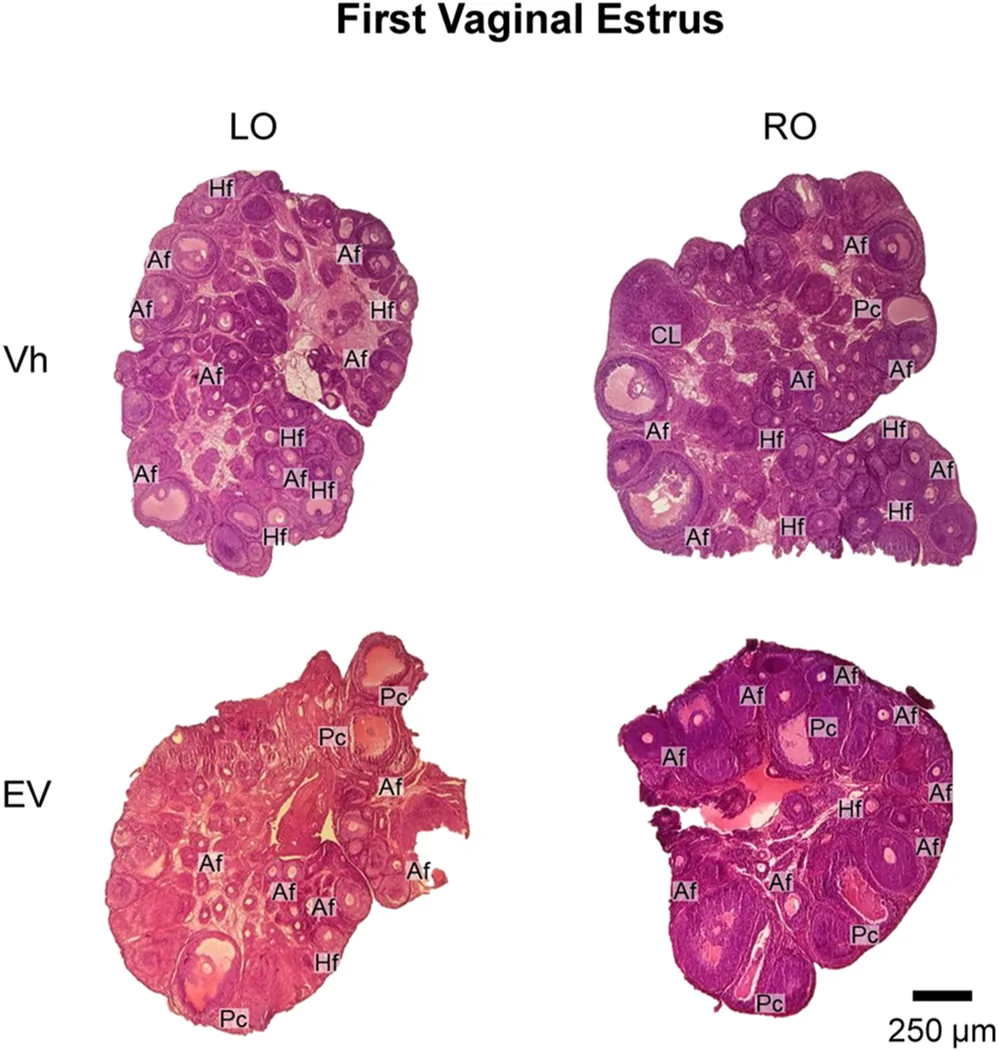
Ovarian histology at the first vaginal estrus in prenatally estrogenized rats. Representative microphotographs of the left (LO) and right (RO) ovaries from offspring exposed on GD 18 to vehicle (Vh) or estradiol valerate (EV) and euthanized at first vaginal estrus. CL, corpora lutea; Hf, healthy follicle; Af, atretic follicle; Pc, precyst; C, cyst. Hematoxylin–eosin staining. Magnification: 4×. The scale bar measures 0.9 cm, which is equivalent to 250 μm. *n* = 6 ovaries per group (3 left and 3 right ovaries from 3 randomly selected rats per cohort).

In both the left and right ovaries of Vh-exposed offspring euthanized at FVE were observed corpora lutea, healthy follicles at various developmental stages, several atretic follicles, and some precysts. The ovarian histology of EV-exposed offspring at FVE was similar to that of the Vh group, although a marked reduction in the number of corpora lutea was evident (Fig. 3).

**Figure 4:**
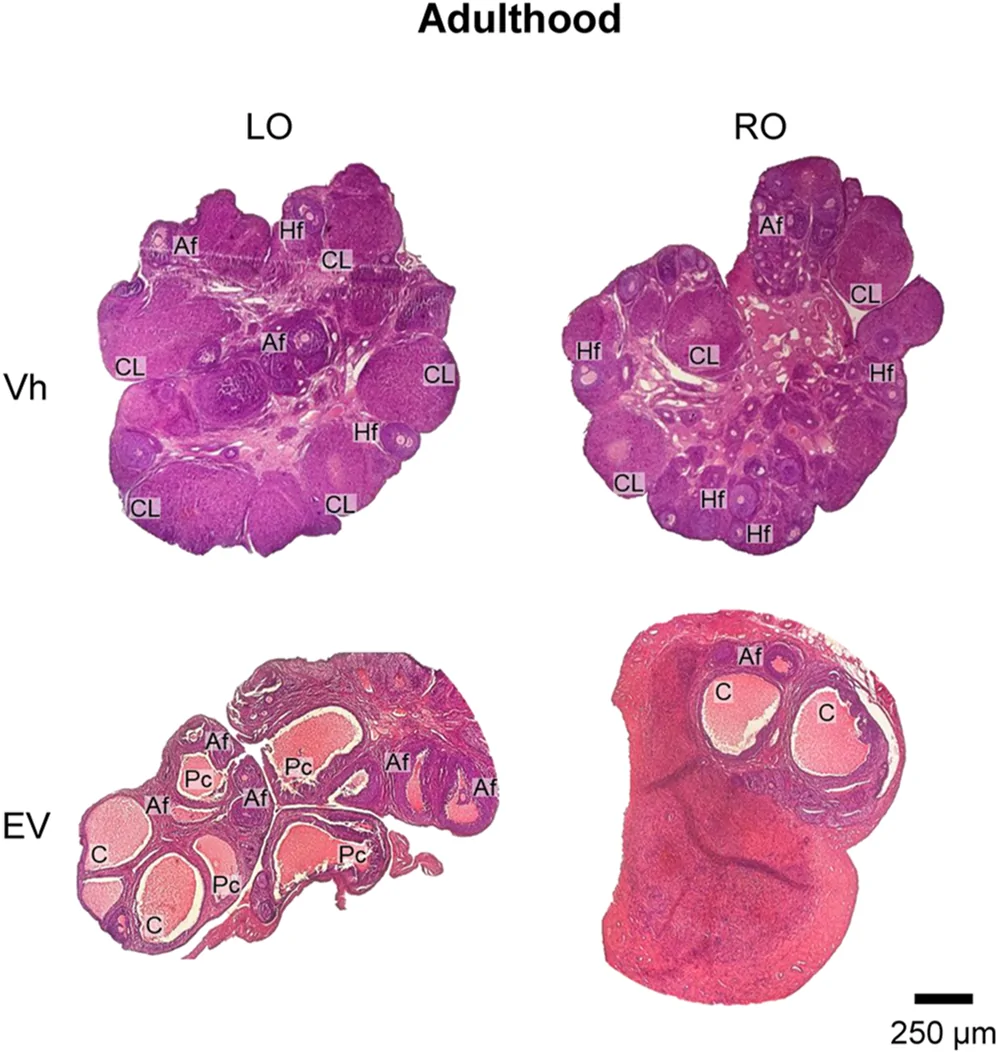
Ovarian histology in adulthood in prenatally estrogenized rats. Representative microphotographs of the left (LO) and right (RO) ovaries from offspring exposed on GD 18 to vehicle (Vh) or estradiol valerate (EV) and euthanized in adulthood, during the estrus stage. CL, corpora lutea; Hf, healthy follicle; Af, atretic follicle; Pc, precyst; C, cyst. Hematoxylin–eosin staining. Magnification: 4×. The scale bar measures 0.9 cm, which is equivalent to 250 μm. *n* = 6 ovaries per group (3 left and 3 right ovaries from 3 randomly selected rats per cohort).

By adulthood, the ovaries of Vh-exposed offspring continued to show corpora lutea, healthy follicles, and atretic follicles; however, the ovaries of EV-exposed offspring exhibited atretic follicles and precysts, alongside the presence of cysts (Fig. 4).

### Follicular population

In Vh-exposed offspring euthanized at FVE, the number of healthy follicles was higher in the RO than in the LO. Compared with their respective Vh groups, EV-exposed offspring showed a higher number of total follicles in the LO, with a large population of atretic follicles. In the RO, these animals exhibited fewer healthy follicles alongside an increase in atretic follicles and precysts and the presence of a cyst (Table 1).

**Table 1:** Follicular dynamics in prenatally estrogenized rats. Median and interquartile range (25–75th percentiles) of total, healthy, and atretic follicles, as well as precysts and cysts, in the left (LO) and right (RO) ovaries of offspring exposed on GD 18 to vehicle (Vh) or estradiol valerate (EV) and euthanized at the first vaginal estrus (FVE) or in adulthood, in estrus. *n* = 6 ovaries per group (3 left and 3 right ovaries from 3 randomly selected rats per cohort).

			Number of follicles				
Groups			Total
		Euthanasia		Healthy	Atretic	Precyst	Cyst
Vh	LO	FVE	85 (82–110)	41 (28–48)	52 (37–69)	2	0
	RO		131 (76–146)	72 (48–94)*	36 (26–74)	1.5 (1–2)	0
EV	LO		123 (115–164)^†^	46 (22–59)	90 (70–103)^†^	3 (2–7)	0
	RO		125 (112–149)	32 (29–43)^†^	89 (78–103)^†^	4 (3–4)^†^	1
Vh	LO	Adulthood	117.5 (68–146)	79 (38.2–122)	23 (19–45)^‡^	3.5 (1–6)	0
	RO		105 (89–118)	71 (50–72)	34 (16–67)	2	0
EV	LO		85 (51–133.3)^‡^	28 (6–50)^†^	46 (26–75)^†^^,^^‡^	11 (4.7–16.5)^‡^	4 (1–7)
	RO		101 (58–129.8)	30.5 (16.2–45.5)^†^	59.5 (36–82.2)^‡^	4 (2–10.5)	1.5 (1–2)

* *P* ≤ 0.05 vs LO.

^†^ *P* ≤ 0.05 vs their respective Vh group.

^‡^ *P* ≤ 0.05 vs their respective FVE group (Mann–Whitney *U* test).

By adulthood, no significant differences in follicular population were observed between the LO and RO of Vh-exposed offspring. However, EV-exposed rats showed a lower number of healthy follicles in both ovaries compared with the Vh group, with an increased atretic follicles population specifically in the LO (Table 1).

When comparing developmental stages, EV-exposed animals in adulthood exhibited a decrease in total and atretic follicles while showing a higher number of precysts and cysts compared with the FVE stage, although some of these differences did not reach statistical significance (Table 1).

When healthy follicles were classified by size, Vh-exposed offspring at FVE showed a higher number of small follicles in the RO than in the LO. In contrast, EV-exposure rats in the RO showed a reduction in the small follicles population compared with the Vh group (Table 2).

**Table 2:** Classification of healthy follicles by size in prenatally estrogenized rats. Median and interquartile range (25–75th percentiles) of small (90–390 μm), medium (391–500 μm), and large (>501 μm) healthy follicles in the left (LO) and right (RO) ovaries of offspring exposed on GD 18 to vehicle (Vh) or estradiol valerate (EV) and euthanized at first vaginal estrus (FVE) or at adulthood, in estrus. *n* = 6 ovaries per group (3 left and 3 right ovaries from 3 randomly selected rats per cohort).

Groups			Number of healthy follicles		
		Euthanasia	Small	Medium	Large
Vh	LO	FVE	41 (28–48)	0	0
	RO		70 (45–93)*	2 (1–3)	2
EV	LO		46 (22–59)	0	0
	RO		32 (28–43)^†^	1	0
Vh	LO	Adulthood	76 (37–118)	1.5 (1–2)	2.5 (2–3)
	RO		66 (50–68)	3.5 (2–5)*	2
EV	LO		27.5 (5.7–49.2)^†^	2	0
	RO		30 (15.2–45.5)^†^	2	0

* *P* ≤ 0.05 vs LO.

^**†**^ *P* ≤ 0.05 vs their respective Vh group (Mann–Whitney *U* test).

By adulthood, Vh-treated rats exhibited a higher number of medium-sized follicles in the RO than in the LO. Conversely, the EV-exposed group showed a lower population of small follicles in both ovaries compared with their respective Vh groups (Table 2).

EV-exposed offspring showed a higher population of small atretic follicles in both the left and right ovaries at puberty and adulthood compared with the Vh group (Table 3).

**Table 3:** Classification of atretic follicles by size in prenatally estrogenized rats. Median and interquartile range (25–75th percentiles) of small (90–390 μm), medium (391–500 μm), and large (>501 μm) atretic follicles in the left (LO) and right (RO) ovaries of offspring exposed on GD 18 to vehicle (Vh) or estradiol valerate (EV) and euthanized at first vaginal estrus (FVE) or at adulthood, in estrus. *n* = 6 ovaries per group (3 left and 3 right ovaries from 3 randomly selected rats per cohort).

Groups			Number of healthy follicles		
		Euthanasia	Small	Medium	Large
Vh	LO	FVE	42 (29–65)	4 (2–10)	6
	RO		29 (24–66)	4.5 (2–7)	4 (1–7)
EV	LO		85 (68–100)*	6	2 (1–3)
	RO		86 (65–101)*	3 (2–6)	7
Vh	LO	Adulthood	17.5 (13–42.2)	2 (1–5.2)	3 (1–4)
	RO		26 (15–63)	4 (1–7)	1
EV	LO		42 (22.2–72.2)*	3 (2–4)	2 (1.2–2.7)
	RO		52 (30–80.7)*	3 (1–7)	1.5 (1–4.2)

* *P* ≤ 0.05 vs their respective Vh group (Mann–Whitney *U* test).

### Steroid hormones

EV-exposed offspring showed lower testosterone levels at FVE compared with the Vh group (EV: 254.0 ± 20.9 vs Vh: 330.0 ± 10.0 pg/mL; *P* ≤ 0.05, Student’s *t*-test). However, by adulthood, these animals displayed reduced progesterone levels (EV: 5.6 ± 0.5 vs Vh: 10.1 ± 0.9 ng/mL; *P* ≤ 0.05, Student’s *t*-test) and an increase in testosterone levels compared with the Vh group (EV: 164.0 ± 11.2 vs Vh: 88.3 ± 18.0 pg/mL; *P* ≤ 0.05, Student’s *t*-test). Serum estradiol levels were not modified at puberty or during adulthood.

### Body and organ weight

To assess the systemic impact of prenatal EV exposure, body and relative organ weights were analyzed. At FVE, EV-exposed offspring showed lower body (EV: 104.2 ± 2.4 vs Vh: 130.4 ± 4.0 g; *P* ≤ 0.05, Student’s *t*-test) and uterine weights (EV: 109.5 ± 8.5 vs Vh: 134.0 ± 4.9 mg/100 g BW; *P* ≤ 0.05, Student’s *t*-test) compared with their respective Vh group. However, by adulthood, EV-exposed animals exhibited an increase in BW (EV: 235.8 ± 6.5 vs Vh: 201.0 ± 2.9 g; *P* ≤ 0.05, Student’s t-test), while the uterine weight remained significantly reduced compared with the Vh group (EV: 113.3 ± 10.4 vs Vh: 155.7 ± 7.0 mg/100 g BW; *P* ≤ 0.05, Student’s *t*-test). No significant differences were observed in ovarian mass.

## Discussion

The results of the present study show that intrauterine exposure to EV on GD 18 affects development, leading to progressive disruptions in reproductive function from puberty to adulthood, culminating in the development of a PCOS-like phenotype. These findings suggest that such disruptions may be driven by the ability of EV, a lipophilic steroid, to readily cross the placental barrier (27).

The onset of puberty is regulated by the reactivation of gonadotropin-releasing hormone (GnRH) neurons and the maturation of the hypothalamic–pituitary–ovarian (HPO) axis (28). Kisspeptin is an indispensable neuropeptide for this process (29) as it directly stimulates GnRH secretion (30). Kiss neurons express estrogen receptors (ER) α, which evidences the regulation of these neurons by estrogens (31). In rodents, these neurons originate during prenatal development, a stage where they already express ERα (GD 11.5–12.5) (32, 33).

Our results show that intrauterine exposure to EV on GD 18 advances VO and first vaginal estrus. This is consistent with findings in rats injected with EV during neonatal (23) or infant stages (19, 20). Interestingly, neonatal exposure to estradiol benzoate (EB) in rats has been shown to decrease GnRH neurons activation and Kiss-immunolabeled fiber density in the anteroventral periventricular and arcuate nuclei; however, it paradoxically accelerates pubertal onset (34). Based on this information, the premature onset of puberty observed in our model raises the possibility that prenatal EV exposure might disrupt Kiss/GnRH signaling or alter the sensitivity of this neuroendocrine pathway. Although central neuroendocrine assessment was not performed in the present study, these findings highlight that early estrogenic insults during critical intrauterine windows can deeply affect the timing of pubertal maturation.

In addition to central mechanisms, we cannot rule out that hormonal stimulation may have exerted a local influence on vaginal tissue. Although some authors propose that puberty occurs in response to high estrogen levels (35), we observed no significant changes in estradiol at puberty. Therefore, rather than a transient hormonal surge, it is possible that prenatal EV exposure sensitized the vaginal epithelium to circulating steroids, similar to what occurs in mice exposed *in utero* to xenoestrogens such as genistein, bisphenol A (BPA), or DES, which exhibit advanced puberty (36, 37).

In the present study, Vh-exposed animals exhibited an asymmetric ovulatory response that shifted with age. At puberty, the right ovary released fewer oocytes than the left ovary, a pattern that reversed in adulthood, where the right ovary became dominant. Although a larger population of healthy follicles was observed in the right ovary at puberty, most were small; this lack of mature follicles explains the lower ovulatory rate compared with adulthood, where the population of medium-sized healthy follicles increases. It has been suggested that these ovulatory asymmetries are indicative of distinct follicular dynamics in each gonad and are associated with differential innervation (38). The right ovary receives a higher density of sympathetic fibers from the superior mesenteric celiac ganglion (39), while the left ovary has a predominance of supraspinal innervation (40). Based on these previous findings, the shift in asymmetry from puberty to adulthood could be related to the differential maturation of these peripheral pathways. Morán *et al.* (41) demonstrated that both the number and connectivity of postganglionic neurons from the celiac-superior mesenteric ganglia to the ovaries undergo dynamic changes during maturation, occurring with a distinct pattern for each ovary.

Prenatal exposure to EV profoundly disrupted the dynamics of follicular development. EV-exposed offspring showed a depletion of small healthy follicles and an increase in atresia at puberty and adulthood, culminating in the development of precysts and cysts. The formation of these structures is a hallmark of EV-induced damage (15, 16, 17, 18, 19, 20, 23). Follicular atresia in this model may be related to FSH, a key gonadotropin in follicular rescue, which decreased in rats injected with EV (15). Our findings also support the timeline described by Cruz *et al.* (23), where the transition from precystic structures to follicular cysts requires a prolonged period after the injection of EV. This explains why follicular cysts were predominantly observed in adulthood rather than at the onset of puberty.

These alterations in the dynamics of follicular development resulted in a blunted ovulatory response at puberty and a complete blockage of ovulation in adulthood, consistent with anovulatory models induced by prepubertal or adult EV injection (15, 16, 17, 18, 19, 20). While the exact mechanisms underlying this anovulation remain to be elucidated in our prenatal design, previous literature allows us to suggest that it might involve both central and peripheral disruptions. Administration of estrogens in infant female rats has been shown to cause alterations at the hypothalamic level, resulting in changes in gonadotropin secretion such as LH, a key hormone in the ovulation process (19). Several research groups, including ours, have demonstrated that EV induces hyperactivity of ovarian sympathetic fibers, as evidenced by an increase in ovarian norepinephrine (NA) levels. This response is directly associated with increased nerve growth factor (NGF) expression (16, 17, 18, 19, 20). Theoretically, NA binds to β-adrenergic receptors in theca cells, stimulating androgen production and creating a hyperandrogenic environment that impairs follicular growth and ovulation (16, 42). Although the direct assessment of these neural and neurotrophic factors was not within the scope of the present study, these literature precedents provide a plausible framework for the progressive reproductive decline observed in our model.

We hypothesize that the loss of ovulation in our model might be linked to early alterations in the ovarian neural environment initiated *in utero*. Evidence indicates that at around GD 16, innervation invades the interior of the mouse’s ovary, forming a dense neural network within the medulla by GD 18 (43). This critical window matches the rapid prenatal development of the sympathetic nervous system and the appearance of catecholaminergic pioneer fibers previously characterized in the fetal rat gonad (21, 22). Since estrogens modulate the expression of neurotrophins, such as nerve growth factor (NGF), which acts as a key signal directing axonal growth (44, 45), prenatal EV exposure could potentially alter the initial patterning of ovarian sympathetic innervation. Although direct quantification of ovarian neural markers was not performed in the present study, our results suggest that the neuroendocrine foundations of PCOS-like dysfunction are highly sensitive to estrogenic insults during critical windows of intrauterine development.

Prenatal exposure to EV altered the estrous cycle pattern, leading to periods of continuous diestrus or persistent estrus. These findings are consistent with models of EV administration during infant or adult stages (16, 19). Similarly, mice prenatally exposed to BPA exhibit altered cycles characterized by prolonged estrus (36), while neonatal administration of zearalenone, a mycoestrogen, results in extended estrus and/or diestrus stages (46, 47). Since the rhythmic functioning of the HPO axis is essential for cycle regularity, the observed acyclicity in our model could suggest an underlying alteration within the regulatory reproductive axis. An imbalanced androgenic or estrogenic environment has been reported to compromise estrous cyclicity (15, 16, 19, 48, 49), as seen in our animals at puberty.

In addition to central disruption, peripheral sympathetic innervation plays a decisive role in regulating the estrous cycle. In rats with EV-induced PCOS, sympathetic hyperactivity of the superior ovarian nerve (SON) is associated with acyclicity. Notably, this condition can be reversed by SON transection (16, 19, 20), showing that this sympathetic innervation plays a determining role in the regulation of the estrous cycle. Therefore, although not directly quantified in our study, it could be hypothesized that early estrogenic insults on these peripheral neural pathways might be a contributing factor to the progressive loss of cyclicity observed from puberty into adulthood.

Hyperandrogenism, primarily characterized by elevated testosterone levels, is a fundamental diagnostic criterion for PCOS, affecting approximately 75–95% of women with this condition (50, 51, 52). It is considered a central and causal manifestation of the syndrome’s pathophysiology (1). In our model, although testosterone levels were low at puberty, they significantly increased by adulthood, a shift accompanied by a marked reduction in progesterone levels.

These diminished progesterone levels could be a direct consequence of the ovulatory blockage observed in EV-exposed offspring. This is consistent with reports on prenatally estrogenized mice, where the corpora lutea were absent (37). Alternatively, the observed hormonal imbalance might suggest that progesterone serves as a precursor for increased testosterone synthesis in adulthood. The hormonal profile observed in these animals at puberty and adulthood is consistent with the progressive structural deterioration of the ovary, as evidenced by the presence of precysts and follicular cysts in adult animals.

Our results show that offspring prenatally exposed to EV exhibited lower BW at puberty, a trend that was reversed in adulthood, where a significant increase was observed. This initial weight reduction may be linked to the maternal physiological state during gestation; EV-treated dams showed decreased weight gain and signs of reduced calorie intake (data not shown). It has been reported that offspring of mothers with restricted feeding exhibit low birth weight due to a reduced nutrient supply to the fetus (53); in our model, this initial deficit could exert a lasting impact that persists until puberty. This is further supported by findings that prenatal exposure to endocrine disruptors, such as DES or BPA, between GD 11–17 results in reduced BW at puberty (36).

However, this early restriction often triggers a ‘catch-up growth’ phenomenon, a rapid period of accelerated growth that attempts to compensate for intrauterine deprivation. We suggest that this process might have been triggered by the intrauterine estrogenic environment. In this regard, Newbold *et al.* (54, 55) described a clear association between environmental estrogens and the development of obesity. These developmental metabolic alterations might have altered energy homeostasis, a hypothesis consistent with findings where neonatal DES exposure in mice caused an initial weight decrease, followed by a recovery period and final weight gain in adulthood (54). In our study, this adult weight increase occurred despite normal estradiol levels, suggesting that the effect is not due to acute hormonal stimulation but rather due to a permanent modification of metabolic pathways or adipose tissue function (55, 56). In murine models, maternal exposure to xenoestrogens (such as genistein, resveratrol, or BPA) for four consecutive days starting on GD 15 has been shown to increase BW by 16 weeks of age (37), further supporting the hypothesis that late-gestational estrogenic insults lay the foundation for adult metabolic dysfunction.

The proliferation and growth of uterine tissue depend on fluctuations in sex hormones, particularly estrogens (57, 58). The uterus expresses ERα, which is essential for epithelial proliferation, and ERβ, which has been associated with an anti-proliferative function (59, 60). Our results demonstrate that prenatal EV exposure leads to a reduced uterine weight at both puberty and adulthood despite normal serum estradiol levels. This suggests that the reduced uterine weight is not a consequence of current circulating estrogens, but rather a result of early intrauterine disruptions. Such prenatal estrogenization could have altered the capacity for *de novo* synthesis of ER or dysregulated the ERα/ERβ ratio. This hypothesis is consistent with the work of Schönfelder *et al.* (59), who reported that maternal BPA treatment (GD 6–21) decreases uterine epithelial thickness in adult offspring. Paradoxically, this reduction was accompanied by higher ERα and lower ERβ expression, suggesting that the lower uterine weight stems from a complex dysregulation of receptor signaling rather than a simple lack of receptors.

**Figure 5:**
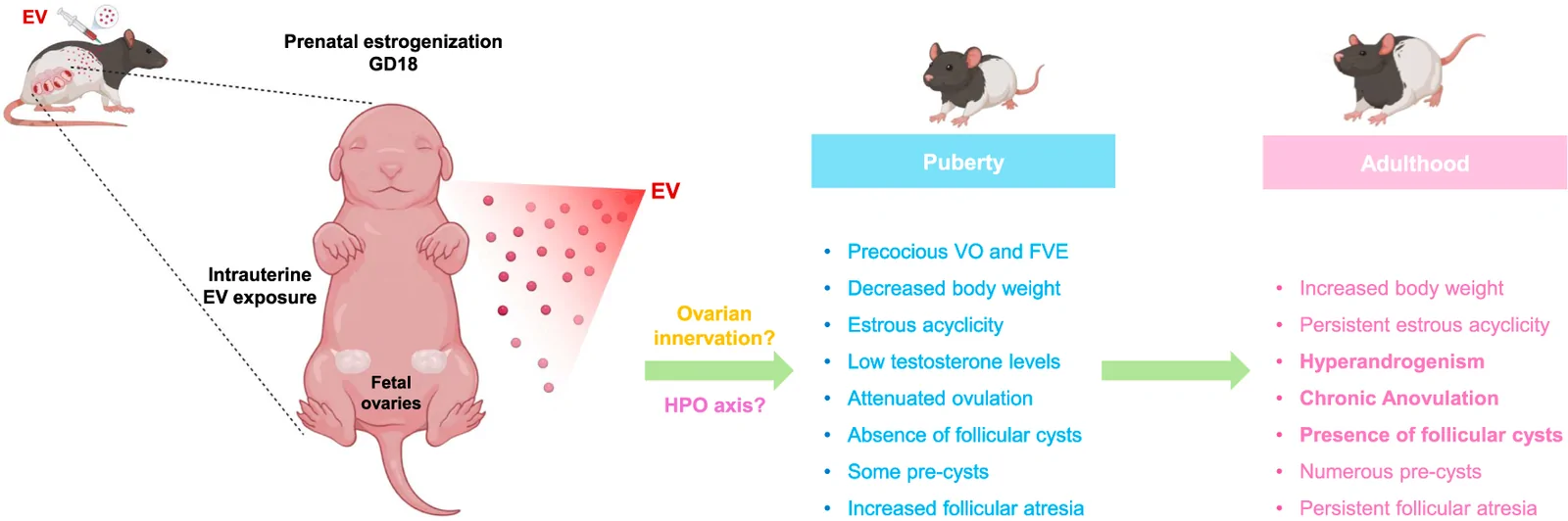
Model of the temporal progression of the reproductive phenotype following prenatal EV exposure. Chronological flow chart illustrating the continuous progression of functional and structural alterations observed postnatally in the offspring following a single intrauterine exposure to estradiol valerate (EV) on gestational day 18 (GD 18). The text in blue (puberty) and pink (adulthood) details the experimentally quantified endpoints at each stage of development. The text accompanied by question marks (ovarian innervation? and HPO axis?) represents the potential neuroendocrine and autonomic pathways hypothesized within the Discussion section as open avenues for future mechanistic validation (Created in BioRender. Gómez-Emeterio, L (2026) https://BioRender.com/5n9aaj3).

Based on our findings, we propose a developmental progression model (Fig. 5), outlining the trajectory of this progressive PCOS-like phenotype. A single intrauterine exposure to EV on GD 18 triggers functional and structural alterations that manifest at puberty and escalate in adulthood. While the potential involvement of altered ovarian innervation and central HPO axis signaling remains framed as a critical open question for future mechanistic validation, this model provides an empirical synthesis of the progressive reproductive deterioration observed in the rat.

### Study limitations

Our results represent a first approach to analyzing the effect of intrauterine EV exposure on the long-term reproductive and ovarian response of the offspring. However, they highlight the necessity of further exploring the mechanisms developing at the hypothalamic level as well as the early stages of fetal ovarian development. Consequently, future research from our group will focus on tracking these early molecular markers (e.g. GDF9 and AMH) and neural components to fully elucidate the ontogeny and precise etiology of this progressive reproductive phenotype.

## Conclusion

Taken together, our findings demonstrate that a single intrauterine exposure of a rat to EV on GD 18 leads to early-onset reproductive disruptions that drive a progressive PCOS-like phenotype. Although the underlying mechanisms responsible for this progressive deterioration remain to be fully determined, these alterations – initiated at puberty – escalate into full endocrine and structural disruptions in adulthood, characterized by increased testosterone levels, follicular cyst formation, and chronic anovulation. Finally, this model supports the Developmental Origins of Health and Disease (DOHaD) hypothesis for PCOS, providing a valuable framework to understand how maternal endocrine imbalances may predispose subsequent generations to reproductive disorders.

## Declaration of interest

The authors declare that there is no conflict of interest that could be perceived as prejudicing the impartiality of the work reported.

## Funding

This research was funded by the Universidad Nacional Autónoma de México under Grant No. UNAM-DGAPA-PAPIIT IN204225.

## Author contribution statement

GR conceived and designed the study; performed investigation and formal analysis; wrote the original draft; and reviewed and edited the manuscript. EMHG performed the methodology, investigation and formal analysis. LGE performed the methodology, investigation and formal analysis, and wrote the original draft of the manuscript. RL, EV, DAR, JAE, and AC reviewed and edited the manuscript. LML conceived and designed the study; supervision and administered the project; acquired funding; and reviewed and edited the manuscript.

## Ethics statement

The experimental protocol was approved by the Research Ethics Committee of the Facultad de Estudios Superiores Zaragoza, Universidad Nacional Autónoma de México (FES-Zaragoza, UNAM; Protocol No. FESZ/CEI/4/3/1/05/24). All procedures were carried out in accordance with the specifications established by the Norma Oficial Mexicana NOM-062-ZOO-1999, technical specifications for the production, care, and use of laboratory animals. Every effort was made to minimize the number of rats used and their suffering.

## AI disclosure

No generative artificial intelligence (AI) tools were used in the preparation of the written or visual content of this manuscript.

## References

[1] Stener-Victorin E, Teede H, Norman RJ, et al. Polycystic ovary syndrome. Nat Rev Dis Primers 2024 10 e27. (10.1038/s41572-024-00511-3) 38637590

[2] Teede HJ, Tay CT, Laven J, et al. Recommendations from the 2023 International evidence-based guideline for the assessment and management of polycystic ovary syndrome. Fertil Steril 2023 120 767–793. (10.1016/j.fertnstert.2023.07.025) 37589624

[3] Helvaci N & Yildiz BO. Polycystic ovary syndrome as a metabolic disease. Nat Rev Endocrinol 2025 21 230–244. (10.1038/s41574-024-01057-w) 39609634

[4] Lucas A. Programming by early nutrition in man. Ciba Found Symp 1991 156 38–55. 1855415

[5] Reynolds LP, Borowicz PP, Caton JS, et al. Developmental programming of fetal growth and development. Vet Clin North Am Food Anim Pract 2019 35 229–247. (10.1016/j.cvfa.2019.02.006) 31103178

[6] Yilmaz B, Terekeci H, Sandal S, et al. Endocrine disrupting chemicals: exposure, effects on human health, mechanism of action, models for testing and strategies for prevention. Rev Endocr Metab Disord 2020 21 127–147. (10.1007/s11154-019-09521-z) 31792807

[7] Varticovski L, Stavreva DA, McGowan A, et al. Endocrine disruptors of sex hormone activities. Mol Cell Endocrinol 2022 539 e111415. (10.1016/j.mce.2021.111415) PMC8762672 34339825

[8] Sir-Petermann T, Maliqueo M, Angel B, et al. Maternal serum androgens in pregnant women with polycystic ovarian syndrome: possible implications in prenatal androgenization. Hum Reprod 2002 17 2573–2579. (10.1093/humrep/17.10.2573) 12351531

[9] Maliqueo M, Lara HE, Sánchez F, et al. Placental steroidogenesis in pregnant women with polycystic ovary syndrome. Eur J Obstet Gynecol Reprod Biol 2013 166 151–155. (10.1016/j.ejogrb.2012.10.015) 23122578

[10] Sir-Petermann T, Codner E, Pérez V, et al. Metabolic and reproductive features before and during puberty in daughters of women with polycystic ovary syndrome. J Clin Endocrinol Metab 2009 94 1923–1930. (10.1210/jc.2008-2836) 19223518 PMC2730345

[11] Abbott DH, Dumesic DA, Eisner JR, et al. Insights into the development of polycystic ovary syndrome (PCOS) from studies of prenatally androgenized female rhesus monkeys. Trends Endocrinol Metab 1998 9 62–67. (10.1016/s1043-2760(98)00019-8) 18406243

[12] Steckler T, Wang J, Bartol FF, et al. Fetal programming: prenatal testosterone treatment causes intrauterine growth retardation, reduces ovarian reserve and increases ovarian follicular recruitment. Endocrinology 2005 146 3185–3193. (10.1210/en.2004-1444) 15802500

[13] Newbold RR, Bullock BC & Mc Lachlan JA. Exposure to diethylstilbestrol during pregnancy permanently alters the ovary and oviduct. Biol Reprod 1983 28 735–744. (10.1095/biolreprod28.3.735) 6850046

[14] Yasuda Y, Kihara T & Nishimura H. Effect of prenatal treatment with ethinyl estradiol on the mouse uterus and ovary. Am J Obstet Gynecol 1977 127 832–836. (10.1016/0002-9378(77)90114-4) 851139

[15] Brawer JR, Munoz M & Farookhi R. Development of the polycystic ovarian condition (PCO) in the estradiol valerate-treated rat. Biol Reprod 1986 35 647–655. (10.1095/biolreprod35.3.647) 3098314

[16] Barria A, Leyton V, Ojeda SR, et al. Ovarian steroidal response to gonadotropins and beta-adrenergic stimulation is enhanced in polycystic ovary syndrome: role of sympathetic innervation. Endocrinology 1993 133 2696–2703. (10.1210/endo.133.6.8243293) 8243293

[17] Lara HE, Ferruz JL, Luza S, et al. Activation of ovarian sympathetic nerves in polycystic ovary syndrome. Endocrinology 1993 133 2690–2695. (10.1210/endo.133.6.7902268) 7902268

[18] Lara HE, Dissen GA, Leyton V, et al. An increased intraovarian synthesis of nerve growth factor and its low affinity receptor is a principal component of steroid-induced polycystic ovary in the rat. Endocrinology 2000 141 1059–1072. (10.1210/endo.141.3.7395) 10698182

[19] Rosa-E-Silva A, Guimaraes MA, Padmanabhan V, et al. Prepubertal administration of estradiol valerate disrupts cyclicity and leads to cystic ovarian morphology during adult life in the rat: role of sympathetic innervation. Endocrinology 2003 144 4289–4297. (10.1210/en.2003-0146) 12960066

[20] Morales-Ledesma L, Linares R, Rosas G, et al. Unilateral sectioning of the superior ovarian nerve of rats with polycystic ovarian syndrome restores ovulation in the innervated ovary. Reprod Biol Endocrinol 2010 8 e99. (10.1186/1477-7827-8-99) PMC2936316 20723258

[21] Champlain JD, Malmfors T, Olson L, et al. Ontogenesis of peripheral adrenergic neurons in the rat: pre- and postnatal observations. Acta Physiol Scand 1970 80 276–288. (10.1111/j.1748-1716.1970.tb04791.x) 5475346

[22] Malamed S, Gibney JA & Ojeda SR. Ovarian innervation develops before initiation of folliculogenesis in the rat. Cell Tissue Res 1992 270 87–93. (10.1007/bf00381883) 1358455

[23] Cruz G, Barra R, González D, et al. Temporal window in which exposure to estradiol permanently modifies ovarian function causing polycystic ovary morphology in rats. Fertil Steril 2012 98 1283–1290. (10.1016/j.fertnstert.2012.07.1060) 22854013

[24] Ramírez Hernández DA, Vieyra Valdez E, Rosas Gavilán G, et al. Role of the superior ovarian nerve in the regulation of follicular development and steroidogenesis in the morning of diestrus 1. J Assist Reprod Genet 2020 37 1477–1488. (10.1007/s10815-020-01787-6) 32363564 PMC7311564

[25] Vieyra E, Ramírez DA, Linares R, et al. Stimulation of nicotinic receptors in the suprachiasmatic nucleus results in a higher number of growing follicles and ova shed. Exp Physiol 2019 104 1179–1189. (10.1113/ep087538) 31241201

[26] Greenwald GG & Roy SK. Follicular development and its control. In The Physiology of Reproduction, vol 2, 2nd edn, pp 629–724. Eds E Knobil & JD Neill. New York: Raven Press, 1994.

[27] Chatuphonprasert W, Jarukamjorn K & Ellinger I. Physiology and pathophysiology of steroid biosynthesis, transport and metabolism in the human placenta. Front Pharmacol 2018 9 e1027. (10.3389/fphar.2018.01027) PMC6144938 30258364

[28] Ebling FJ & Cronin AS. The neurobiology of reproductive development. Neuroreport 2000 11 23–33. (10.1097/00001756-200011090-00002) 11095489

[29] Dungan HM, Clifton DK & Steiner RA. Minireview: kisspeptin neurons as central processors in the regulation of gonadotropin-releasing hormone secretion. Endocrinology 2006 147 1154–1158. (10.1210/en.2005-1282) 16373418

[30] Herbison AE. Control of puberty onset and fertility by gonadotropin-releasing hormone neurons. Nat Rev Endocrinol 2016 12 452–466. (10.1038/nrendo.2016.70) 27199290

[31] Wintermantel TM, Campbell RE, Porteous R, et al. Definition of estrogen receptor pathway critical for estrogen positive feedback to gonadotropin-releasing hormone neurons and fertility. Neuron 2006 52 271–280. (10.1016/j.neuron.2006.07.023) 17046690 PMC6116893

[32] Desroziers E, Droguerre M, Bentsen AH, et al. Embryonic development of kisspeptin neurones in rat. J Neuroendocrinol 2012 24 1284–1295. (10.1111/j.1365-2826.2012.02333.x) 22530935

[33] Kumar D, Freese M, Drexler D, et al. Murine arcuate nucleus kisspeptin neurons communicate with GnRH neurons in utero. J Neurosci 2014 34 3756–3766. (10.1523/jneurosci.5123-13.2014) 24599473 PMC6608990

[34] Bateman HL & Patisaul HB. Disrupted female reproductive physiology following neonatal exposure to phytoestrogens or estrogen specific ligands is associated with decreased GnRH activation and kisspeptin fiber density in the hypothalamus. Neurotoxicology 2008 29 988–997. (10.1016/j.neuro.2008.06.008) 18656497 PMC2647326

[35] Ojeda SR & Skinner MK. Puberty in the rat. In Encyclopedia of Reproduction, vol 2, 3rd ed. pp 2061–2126. Eds E Knobil & JD Neill. San Diego: Academic Press, 2006. (10.1016/B978-012515400-0/50043-9)

[36] Honma S, Suzuki A, Buchanan DL, et al. Low dose effect of in utero exposure to bisphenol A and diethylstilbestrol on female mouse reproduction. Reprod Toxicol 2002 16 117–122. (10.1016/s0890-6238(02)00006-0) 11955942

[37] Nikaido Y, Yoshizawa K, Danbara N, et al. Effects of maternal xenoestrogen exposure on development of the reproductive tract and mammary gland in female CD-1 mouse offspring. Reprod Toxicol 2004 18 803–811. (10.1016/j.reprotox.2004.05.002) 15279878

[38] Domínguez R & Cruz-Morales SE. The ovarian innervation participates in the regulation of ovarian functions. Endocrinol Metabol Syndr 2011 4.

[39] Klein CM & Burden HW. Anatomical localization of afferent and postganglionic sympathetic neurons innervating the rat ovary. Neurosci Lett 1988 85 217–222. (10.1016/0304-3940(88)90354-0) 3374837

[40] Tóth IE, Wiesel O, Boldogkoi Z, et al. Predominance of supraspinal innervation of the left ovary. Microsc Res Tech 2007 70 710–718. (10.1002/jemt.20456) 17393475

[41] Morán C, Zarate F, Morán JL, et al. Lateralization of the connections of the ovary to the celiac ganglia in juvenile rats. Reprod Biol Endocrinol 2009 7 e50. (10.1186/1477-7827-7-50) PMC2697162 19460167

[42] Luna SL, Neuman S, Aguilera J, et al. In vivo β-adrenergic blockade by propranolol prevents isoproterenol-induced polycystic ovary in adult rats. Horm Metab Res 2012 44 676–681. (10.1055/s-0031-1301304) 22328164

[43] McKey J, Bunce C, Batchvarov IS, et al. Neural crest-derived neurons invade the ovary but not the testis during mouse gonad development. Proc Natl Acad Sci U S A 2019 116 5570–5575. (10.1073/pnas.1814930116) 30819894 PMC6431225

[44] Toran-Allerand CD. Mechanisms of estrogen action during neural development: mediation by interactions with the neurotrophins and their receptors? J Steroid Biochem Mol Biol 1996 56 169–178. (10.1016/0960-0760(95)00234-0) 8603038

[45] Dissen GA, Romero C, Paredes A, et al. Neurotrophic control of ovarian development. Microsc Res Tech 2002 59 509–515. (10.1002/jemt.10227) 12467027

[46] Hilakivi-Clarke L, Cho E & Clarke R. Maternal genistein exposure mimics the effects of estrogen on mammary gland development in female mouse offspring. Oncol Rep 1998 5 609–616. (10.3892/or.5.3.609) 9538161

[47] Nikaido Y, Yoshizawa K, Pei RJ, et al. Prepubertal zearalenone exposure suppresses N-methyl-N-nitrosourea-induced mammary tumorigenesis but causes severe endocrine disruption in female Sprague-Dawley rats. Nutr Cancer 2003 47 164–170. (10.1207/s15327914nc4702_9) 15087269

[48] Mannerås L, Cajander S, Holmäng A, et al. A new rat model exhibiting both ovarian and metabolic characteristics of polycystic ovary syndrome. Endocrinology 2007 148 3781–3791. (10.1210/en.2007-0168) 17495003

[49] Osuka S, Iwase A, Nakahara T, et al. Kisspeptin in the hypothalamus of 2 rat models of polycystic ovary syndrome. Endocrinology 2017 158 367–377. (10.1210/en.2016-1333) 27983870

[50] Waldman IN & Legro RS. Polycystic ovary syndrome. In The Ovary, 3rd edn. pp 415–435. Eds PCK Leung & EY Adashi. United Kingdom: Academic Press, 2019. (10.1016/B978-0-12-813209-8.00026-1)

[51] Witchel SF, Oberfield SE & Peña AS. Polycystic ovary syndrome: pathophysiology, presentation, and treatment with emphasis on adolescent girls. J Endocr Soc 2019 3 1545–1573. (10.1210/js.2019-00078) 31384717 PMC6676075

[52] Stener-Victorin E & Deng Q. Epigenetic inheritance of polycystic ovary syndrome - challenges and opportunities for treatment. Nat Rev Endocrinol 2021 17 521–533. (10.1038/s41574-021-00517-x) 34234312

[53] Woodall SM, Breier BH, Johnston BM, et al. A model of intrauterine growth retardation caused by chronic maternal undernutrition in the rat: effects on the somatotrophic axis and postnatal growth. J Endocrinol 1996 150 231–242. (10.1677/joe.0.1500231) 8869590

[54] Newbold RR, Padilla-Banks E, Snyder RJ, et al. Developmental exposure to endocrine disruptors and the obesity epidemic. Reprod Toxicol 2007 23 290–296. (10.1016/j.reprotox.2006.12.010) 17321108 PMC1931509

[55] Newbold RR, Padilla-Banks E & Jefferson WN. Environmental estrogens and obesity. Mol Cell Endocrinol 2009 304 84–89. (10.1016/j.mce.2009.02.024) 19433252 PMC2682588

[56] Hatch EE, Troisi R, Palmer JR, et al. Prenatal diethylstilbestrol exposure and risk of obesity in adult women. J Dev Orig Health Dis 2015 6 201–207. (10.1017/s2040174415000033) 25697972

[57] Katzenellenbogen JA, Johnson HJ Jr & Carlson KE. Studies on the uterine, cytoplasmic estrogen binding protein. Thermal stability and ligand dissociation rate. An assay of empty and filled sites by exchange. Biochemistry 1973 12 4092–4099. (10.1021/bi00745a011) 4745661

[58] Wall EH, Hewitt SC, Case LK, et al. The role of genetics in estrogen responses: a critical piece of an intricate puzzle. FASEB J 2014 28 5042–5054. (10.1096/fj.14-260307) 25212221 PMC4232287

[59] Schönfelder G, Friedrich K, Paul M, et al. Developmental effects of prenatal exposure to bisphenol a on the uterus of rat offspring. Neoplasia 2004 6 584–594. (10.1593/neo.04217) 15548368 PMC1531663

[60] Zama AM & Uzumcu M. Epigenetic effects of endocrine-disrupting chemicals on female reproduction: an ovarian perspective. Front Neuroendocrinol 2010 31 420–439. (10.1016/j.yfrne.2010.06.003) 20609371 PMC3009556

